# Eliminating Spiked Bovine Spongiform Encephalopathy Agent Activity from Heparin

**DOI:** 10.3201/eid2610.200142

**Published:** 2020-10

**Authors:** Cyrus Bett, Omozusi Andrews, David M. Asher, Teresa Pilant, David Keire, Luisa Gregori

**Affiliations:** US Food and Drug Administration, Silver Spring, Maryland, USA (C. Bett, O. Andrews, D.M. Asher, T. Pilant, L. Gregori);; US Food and Drug Administration, St. Louis, Missouri, USA (D. Keire)

**Keywords:** bovine spongiform encephalopathy, bovine heparin, porcine heparin, heparin, prion protein, prions

## Abstract

US manufacturers, concerned about bovine spongiform encephalopathy (BSE), ceased marketing bovine heparin in the 1990s. Recent short supplies of safe porcine heparin suggest that reintroducing bovine heparin might benefit public health. We purified heparin from crude bovine extract spiked with BSE agent, removing substantial infectivity and abnormal prion proteins (PrP^TSE^).

Heparin is a widely used injectable anticoagulant. In the 1990s, bovine-derived heparin was withdrawn from the US market because of concerns about possible contamination of bovine tissues with the agent of bovine spongiform encephalopathy (BSE), the causative agent of variant Creutzfeldt-Jakob disease (vCJD) in humans (*1*). Currently, only porcine heparin, mostly from China, is marketed in the United States. The US Food and Drug Administration has encouraged reintroduction of bovine-sourced heparin into the US market to improve the reliability of the heparin supply chain by diversifying sources (*2*,*3*). The risk that BSE agent might contaminate bovine tissues is now very small because of safeguards implemented during the BSE crisis (*4*). 

We previously showed that a model 4-step bench-scale heparin manufacturing process cleared substantial amounts of spiked scrapie agent, a surrogate for BSE agent (*5*). Our protocol yielded heparin with physicochemical identity, purity, and potency similar to those of United States Pharmacopeia (USP) standard heparin. In this study, we spiked commercial crude bovine heparin with BSE agent itself and processed samples using the same manufacturing process we applied to scrapie agent. We tested each intermediate product for residual abnormal prion protein (PrP^TSE^, a biochemical marker of BSE) and infectivity. We assayed BSE infectivity using intracerebral inoculations of 30-μL volumes into BSE-susceptible transgenic mice (TgBo110) overexpressing the bovine prion-protein–encoding (*PRNP*) gene (*6*). To overcome heparin’s acute toxicity when administered intracerebrally into mice, we diluted the samples; 10^−4^ was the lowest dilution tolerated. 

We ended the study 2 years after inoculations, testing brains of all mice for PrP^TSE^ using the HerdCheck BSE-Scrapie Ag Test (IDEXX Laboratories, https://www.idexx.com) (*7*), which was previously found to be more sensitive than Western blots (*8*), to assign final disease status (Table). We detected infectivity in samples up to the diatomaceous-earth (DE) filtration step. We estimated removals by DE filtration conservatively, assuming that a 10-fold lower dilution, not tested, would have infected all mice. Sodium hydroxide (NaOH) treatment removed 1.7 log_10_ of BSE infectivity and DE filtration removed >1.1 log_10_ of BSE infectivity. To increase sensitivity of the mouse bioassay, we removed heparin by centrifuging samples (20,000 × *g*, 1 hr, 4°C), washed the pellets, resuspended them in inoculation buffer, and inoculated mice as described. We tested brains of all mice for PrP^TSE^ as reported previously (5). We detected residual infectivity in all aliquots, including the final product. NaOH treatment removed 1.5 log_10_ of BSE infectivity. We estimated removals by other steps. DE filtration removed ≥1.4 log_10_ of BSE infectivity. The hydrogen peroxide bleaching and methanol precipitation (final product) steps each removed <1 log_10_ of infectivity, considered negligible. Thus, cumulatively, scaled-down heparin purification removed a total of >2.9 log_10_ of BSE infectivity; NaOH treatment and DE filtration were the only effective steps.

**Table:** Reduction of BSE agent measured by animal infectivity bioassay and PrP^TSE^ amplification with RT-QuIC*

Brain dilutions	Samples with heparin						Samples after heparin removed				
	BSE spike	NaOH treatment	DE filtration	H_2_O_2_ bleaching	Final product		BSE spike	NaOH treatment	DE filtration	H_2_O_2_ bleaching	Final product
10^−1^	NT	NT	NT	NT	NT		NT	NT	NT	NT	3/5
10^−2^	NT	NT	NT	NT	NT		5/5	5/5	2/4	2/5	0/5
10^−3^	NT	NT	NT	NT	NT		5/5	3/4	2/4	0/5	0/5
10^−4^	19/19	16/16	3/19	0/19	0/20		4/4	3/5	0/5	0/5	0/5
10^−5^	8/8	3/10	0/10	0/10	0/10		2/4	0/5	0/5	0/5	0/5
10^−6^	9/9	0/10	0/10	0/10	0/10		2/5	0/5	0/5	0/5	NT
10^−7^	0/5	NT	NT	NT	NT		0/5	NT	NT	NT	NT

Sample	Animal bioassay								RT-QuIC		
	Samples with heparin				Samples after heparin removed				Samples after heparin removed		
	log_10_ ID_50_/g brain	log_10_ removal			log_10_ ID_50_/g brain	log_10_ removal			log_10_ SD_50_/g brain	log_10_ removal	
		Step	Total			Step	Total			Step	Total
BSE spike	8.0	NA	NA		6.9	NA	NA		9.0 ± 0.2	NA	NA
NaOH treatment	6.3	1.7	1.7		5.4	1.5	1.5		6.6 ± 0.3	2.4	2.4
DE filtration	<5.2	>1.1	>2.8		<4.0	>1.4	>2.9		5.3 ± 0.1	1.3	3.7
H_2_O_2_ bleaching	ND	NA	NA		<3.4	>0.6	>3.5		5.0 ± 0.4	0.3	4.0
Final product	ND	NA	NA		<2.6	>0.8	>4.3		5.0 ± 0.6	0.0	4.0

*Values are the number of animals with confirmed BSE divided by the number of animals inoculated. BSE, bovine spongiform encephalopathy; DE diatomaceous earth; H_2_O_2_, hydrogen peroxide; ID_50_, 50% infectious dose; NA, not applicable; NaOH, sodium hydroxide; ND, not detectable; NT, not tested; RT-QuIC, real-time quaking-induced conversion; SD_50_, 50% seeding dose; TSE, transmissible spongiform encephalopathies.

We also quantified residual PrP^TSE^ in each sample using the real-time quaking-induced conversion (RT-QuIC) assay with hamster–sheep chimeric prion protein (*9*) as substrate, expressing results as log_10_ 50% seeding doses (SD_50_), as reported previously (*5*). We detected PrP^TSE^ in unspun BSE spike and NaOH-treated samples but only inconsistent signals in aliquots from successive steps (data not shown). To increase sensitivity and remove heparin interfering with RT-QuIC at low concentrations of PrP^TSE^, we centrifuged all samples as we did previously. To quantify PrP^TSE^, we resuspended pellets and serially diluted each sample in phosphate-buffered saline 0.05% sodium dodecyl sulfate, adding 2 µL of each dilution to seed RT-QuIC, each dilution into quadruplicate wells (see log_10_ SD_50_ values in Table). NaOH treatment removed 2.4 log_10_ of PrP^TSE^ and DE filtration steps removed 1.3 log_10_ of PrP^TSE^. Hydrogen peroxide bleaching and methanol precipitation reduced PrP^TSE^ by only negligible amounts. Thus, processing from crude heparin to final pharmaceutical heparin cumulatively removed 3.7 log_10_ of spiked PrP^TSE^.

We showed previously, using a rodent-adapted scrapie agent, that heparin processing removed 3.6 log_10_ of scrapie infectivity and 3.4 log_10_ of PrP^TSE^ (*5*). Here, we report studies with the more relevant BSE agent itself, showing similar reduction by 3.7 log_10_ of PrP^TSE^. We could demonstrate only >2.9 log_10_ reduction in infectivity, because the starting titer of the BSE-infected brain homogenate was low. However, we detected both residual BSE infectivity and PrP^TSE^ seeding activity after final steps of processing, so our model process did not yield sterile heparin. We found NaOH treatment and DE filtration to be the most effective steps for removing both BSE infectivity and PrP^TSE^ seeding activity, consistent with previous results using scrapie agent.

Overall, our data suggest that typical heparin manufacturing is likely to remove substantial amounts of BSE agent. Furthermore, a probabilistic model assessing the vCJD risk for bovine heparin sourced from cattle in the United States and Canada estimated the risk to be very low (*10*). The demonstrated ability of a typical heparin purification process to remove substantial amounts of contaminating BSE agent, taken together with careful selection of low-risk bovine material to manufacture heparin, provides additional assurance of safety, supporting eventual reintroduction of bovine heparin to the US market.
